# Stitching brain and spinal cord DTI using cross-correlation registration: toward an atlas of spinal tracts

**DOI:** 10.1186/s41747-025-00647-1

**Published:** 2025-11-14

**Authors:** Corentin Dauleac, Amine Boukhari, Timothée Jacquesson, Guillaume Criton, François Cotton, Carole Frindel

**Affiliations:** 1 Hospices Civils de Lyon, Hôpital neurologique et neurochirurgical Pierre Wertheimer, Service de Neurochirurgie, Lyon, France; 2 Université Lyon I, Université Claude Bernard, Lyon, France; 3 Laboratoire CREATIS, CNRS UMR5220, Inserm U1206, INSA-Lyon, Université de Lyon I, Lyon, France; 4 Hospices Civils de Lyon, Centre Hospitalier de Lyon Sud, Service de Radiologie, Lyon, France

**Keywords:** Diffusion tensor imaging, Spinal cord, Stitching, Tractography, White matter

## Abstract

**Background:**

Robust and continuous *in vivo* differentiation of spinal tracts along the brain–spinal cord axis is limited. We stitched brain and spinal cord diffusion tensor imaging (DTI) to create continuity between the brain and cervical spinal cord, enabling tractography along the central nervous system, producing an atlas of the spinal cord white matter.

**Materials and methods:**

This prospective pilot study included four healthy subjects. Brain and cervical spinal cord 3-T DTI acquisitions were performed. Distortions were corrected using the Functional magnetic resonance imaging of the brain Software Library (FSL) software package. A semiautomatic stitching process was achieved using cross-correlation. Once the highest correlation peak was identified, rigid registration allowed accurate image alignment and fusion. Regions of interest were drawn in the brainstem according to atlas-guided projection tracts. Fiber tracking was performed using a deterministic approach with Diffusion Spectrum Imaging (DSI) Studio.

**Results:**

The median fiber length from stitched-image tractography (192 mm) was significantly greater than that from both the brain (111.5 mm) and spinal cord (115 mm) fields of view. The white matter fiber atlas described: the corticospinal tract in the medial part of the lateral funiculus; the rubrospinal tract in the lateral funiculus, overlapped with the corticospinal tract; the gracilis and cuneatus tracts in the dorsal columns; the spinothalamic tract in the ventrolateral part of the spinal cord, around the ventral horn; and the spinocerebellar tracts overlapping them, in the lateral funiculus.

**Conclusion:**

Stitching brain and spinal cord DTI fields of view provided an *in vivo* spinal cord white matter atlas in humans.

**Relevance statement:**

This study provides a detailed and individualized mapping of spinal tracts, serving as a potential tool for neurosurgical planning, particularly in procedures involving intramedullary tumors. It also may enhance the accuracy of prognostic assessments in patients with spinal cord injury, multiple sclerosis, or degenerative myelopathy.

**Key Points:**

- Brain and spinal cord diffusion tensor imaging scans were stitched to map spinal tracts across the central nervous system, enabling detailed three-dimensional imaging of spinal cord pathways.
- The resulting images showed precise locations of spinal tracts, producing an *in vivo* atlas of the spinal cord white matter.
- This tool may help surgeons plan safer operations and better predict outcomes in spinal cord disorders.

**Graphical Abstract:**

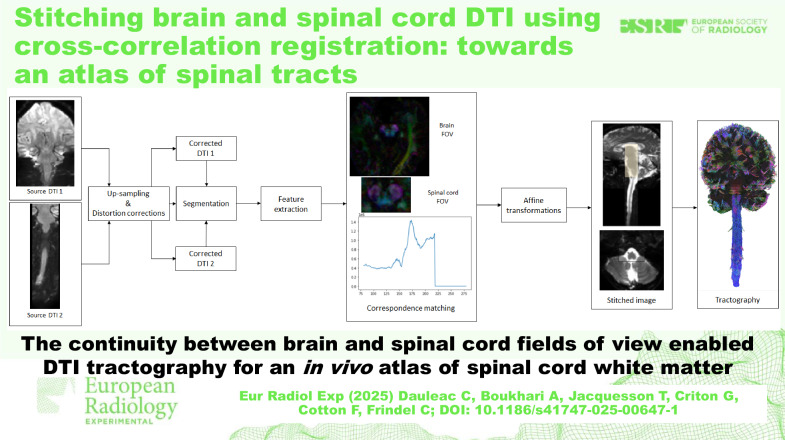

## Background

In the last decade, diffusion tensor imaging (DTI) and tractography have emerged as powerful tools for exploring the microstructural architecture of the intricate neural pathways within the spinal cord [1]. Spinal cord tractography allows for the visualization and analysis of white matter fibers, offering valuable insights into the organization of the spinal tracts [2]. Recent advances in DTI protocols [3, 4] and postprocessing techniques [5–7] have significantly enhanced the understanding of the spinal cord’s microstructural organization and the ability to track neural fibers *in vivo*, both in normal [1, 2] and pathological conditions [8–10]. These advancements have enabled researchers and clinicians to investigate various spinal cord diseases, such as spinal cord injury [11, 12], spinal cord tumors [9, 13–15], and multiple sclerosis [16, 17]; contributing to improved diagnostic and therapeutic strategies [9, 14, 15].

Despite these advancements, current spinal cord tractography faces some limitations; the most important is the inability to differentiate spinal tracts from each other [3]. This limitation is due to the complex anatomy of the spinal cord [18] and that magnetic resonance imaging (MRI) machine manufacturers do not offer machines with a field of view (FOV) that includes the entire central nervous system. This prevents the acquisition of continuous imaging from the brain to the spinal cord. As a result, it is challenging to accurately identify and delineate spinal projection tracts, which hampers comprehensive mapping and understanding of spinal cord anatomy.

To address these challenges, the stitching of brain and spinal cord DTI through cross-correlation registration methodology [19] presents an innovative and promising solution. Image stitching technology has been successfully applied in various imaging contexts [20, 21] (especially using anatomical images), but to the best of our knowledge, no previous study has demonstrated a fully integrated DTI stitching pipeline between the brain and spinal cord FOV *in vivo*. This stitching approach could allow for the seamless alignment and merging of brain and spinal cord imaging data, facilitating the continuous tracking of neural fibers from the brain to the spinal cord.

Therefore, the present study aimed to provide a proof of concept for stitching DTI data from the brain to the spinal cord, focusing on developing a methodology to differentiate and map spinal tracts *in vivo*, ultimately contributing to the creation of a comprehensive and accurate atlas of spinal cord white matter.

## Materials and methods

### Demographics

Four healthy volunteers (two women and 2 men, mean age: 32.4 years) were included in this prospective, single-center study. All procedures were approved by an institutional review board (*Comité de Protection des Personnes Sud-Méditerranée II*, 2021-A02277-34) and by the national data protection committee (*Commission nationale de l’informatique et des libertés*, MR0001, 10/28/2021), and were conducted in accordance with the 1964 Helsinki declaration and its later amendments or comparable ethical standards. Written informed consent was obtained from all participants.

The inclusion criteria were: age between 18 and 50 years, no previous neurological or neurosurgical history at the brain, spine and spinal cord levels, no previous history of brain or neck surgery, and no MRI contraindication. Moreover, in order to minimize acquisition artifacts, subjects with dental implants or braces were excluded.

### Data acquisition

Subjects were positioned with the neck in a neutral position to have the cervical spinal cord as straight as possible, in the axis of the brainstem. If cervical lordosis was seen on the initial survey, a cushion was placed under the subject’s head, or the subject was asked to tilt his/her head toward his/her chest, to standardize cervical lordosis angles. Brain and cervical spinal cord diffusion images were acquired during the same imaging session, using a 3-T Ingenia Elition MRI machine (Philips Medical System) with a 32-channel coil.

#### Brain diffusion imaging

Brain diffusion images were acquired with the following parameters: 32 directions; *b*-value 1,000 s/mm^2^; echo time 91 ms, repetition time 7,038 ms; isotropic voxel size 2 × 2 × 2 mm^3^, no slice gap; FOV 224 × 184 × 200 mm^3^, with *B*0 opposed phase-encoding directions, without in-plane acceleration: Acquisition time was 8:12 min:s. The acquisition box included the brain from the convexity (upper part) to the medulla (lower part).

#### Spinal cord diffusion imaging

Spinal cord diffusion images were acquired using zonally magnified oblique multislice (ZOOM) echo planar imaging. These methods exploit the concept of inner volume imaging [22–24] and enable the selective excitation and refocusing of a reduced FOV (224 × 56 × 40 mm^3^). The following parameters were used: *b*-value: 1,000 s/mm^2^, echo time 64 ms, repetition time 3,220 ms; isotropic voxel size 2 × 2 × 2 mm^3^, no slice gap. The acquisition box included the whole brainstem to the C_7_ spine level and was oriented in the axis of the spinal cord (according to the sagittal plane). The sequence was first acquired with the right-left phase-encoding direction in 3:45 min:s, and second with the left-right phase-encoding direction (to perform distortion corrections, which can be increased at the extremities of the acquisition field) in 3:45 min:s, without in-plane acceleration, with a total of 64 directions.

#### Anatomical imaging

Brain T2-weighted imaging was acquired using a three-dimensional spin-echo sequence with the following parameters: FOV 250 × 180 × 250 mm^3^, voxel size 1 × 1 × 1 mm, no slice gap; echo time 280 ms, repetition time 3,000 ms. Acquisition time was 2:10 min:s. Spinal cord T2-weighted imaging was acquired using a turbo spin-echo sequence, with the following parameters: FOV 300 × 31 × 160 mm^3^, voxel size 0.8 × 0.95 × 2 mm^3^, no slice gap; sagittal plane, echo time 120 ms, repetition time 3,500 ms. Acquisition time was 2:27 min:s. In order to differentiate gray matter from white matter, axial T2*-weighted imaging was added using fast field-echo, focused on each spinal level (C_1_, C_2_, C_3_, C_4_, C_5_, C_6,_ and C_7_), with the following parameters: FOV 3 × 150 × 150 mm^3^; voxel size 0.9 × 0.7 × 3 mm^3^, echo time 9.2 ms, repetition time 255 ms, fat suppression; Acquisition time was 1:50 min:s. T2-weighted acquisitions were chosen due to their superior contrast for differentiating the spinal cord from surrounding cerebrospinal fluid, and T2*-weighted images were particularly effective for segmenting gray matter *versus* white matter—both essential for accurate anatomical alignment and subsequent registration.

### Data processing

**Fig. 1:**
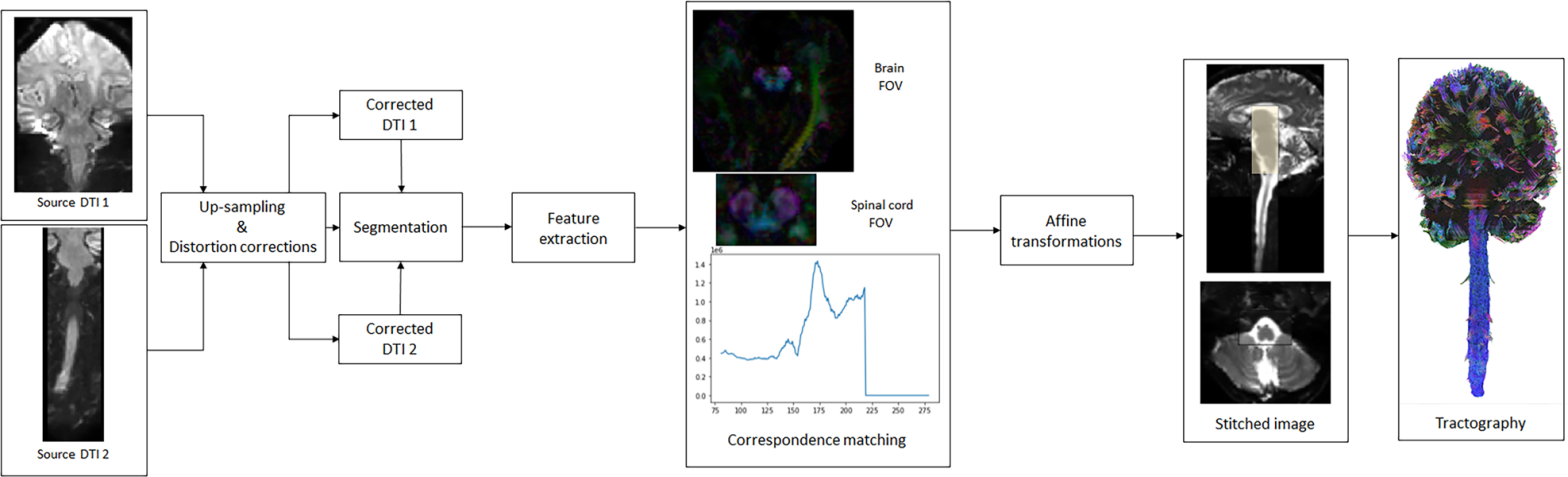
Overview of the template-based analysis pipeline. Brain and spinal cord diffusion tensor images were acquired in a coronal plane, with two different fields of view. Upsampling 2 was performed, and distortions were corrected in order to minimize field inhomogeneity, eddy current, and motion artifacts. Images were segmented to eliminate anything that was not part of the nervous system on images. Feature extraction, such as pixel intensity, was performed before to achieve cross-correlation, where the highest correlation peak was identified and indicated the best overlap. Affine transformations were performed and images were stitched (yellow area corresponds to the stitched overlap) before to obtain brain and spinal cord tractography according to a deterministic approach

The data processing workflow is illustrated in Fig. 1.

#### Distortion corrections

Upsampling by 2 was achieved uniformly across the three spatial dimensions (*x*, *y*, *z*) to improve spatial resolution and ensure consistent scaling between brain and spinal cord volumes. Then, distortion corrections using opposed phase-encoding directions were intended to correct for geometric distortions that arise primarily from B_0_ inhomogeneities and susceptibility-induced distortions. These distortions tend to cause stretching, compression, or shearing of the image, especially in the phase-encoding direction. When applying these corrections, the algorithm combines information from both phase-encoding directions to estimate the underlying distortions and applies a warp field to correct the images. Therefore, the reduction of inhomogeneity distortions [25] was performed using Functional magnetic resonance imaging of the brain Software Library (FSL) [26] topup [27], implemented in Diffusion Spectrum Imaging (DSI) Studio (version Aug 2022, http://dsi-studio.labsolver.org). Artifacts generated by eddy currents produced during diffusion-encoding gradient application were corrected using FSL eddy implemented in DSI Studio.

Due to subject motion, residual misalignment may still occur between brain and spinal cord acquisitions, particularly at the stitching interface. To mitigate this, subjects were instructed to remain as still as possible, and head positioning was standardized using cushions when necessary. Moreover, each diffusion-weighted image was linearly registered to the *B*0 using mutual information to correct motion artifacts.

#### Stitching

The stitching process is a multistep procedure designed to align and integrate brain and spinal cord images. This approach ensured precise mapping of neural structures by overcoming potential sources of noise and misalignment. The five steps involved are described in the following paragraphs.

1. Initially, a mask was defined to encompass both the brain and the spinal cord while excluding non-nervous system structures, which could introduce noise into the images. This mask was generated semi-automatically using DSI Studio, which applies an intensity-based thresholding method. The threshold could be manually adjusted to account for inter-subject variability, ensuring adequate coverage of relevant anatomical features while maintaining a standardized procedure. This step helped to enhance the accuracy of subsequent image processing steps by focusing on relevant anatomical features.
2. Then, a reference image with a distinctive and easily identifiable shape—corresponding to “Mickey Mouse head” of the midbrain (in the axial plane, Fig. 1)—was chosen in the brain volume. This reference image was used to calculate the cross-correlation with all axial images in the spinal cord volume. For each spinal slice $B\left(x,y,z\right)$, a two-dimensional cross-correlation score.
3. ${CC}\left(z\right)$ was calculated as:
  $$\sum {CC}\left(z\right)={\sum }_{x}{\sum }_{y}A\left(x,y\right)B(x,y,z)$$
  where $A\left(x,y\right)$ is the intensity of the reference image and $B\left(x,y,z\right)$ is the intensity of the spinal cord volume at slice *z*. This step measured the degree of similarity between the reference image and each image in the spinal cord volume to estimate their axial alignment and determine the region of overlap between the two volumes.
4. Next, axial registration of each matched image pair (each image in the brain stack with its corresponding image in the spinal cord volume) was refined using cross-correlation, now accounting for bother translation and in-plane rotation. The cross-correlation in this case was defined as:
  $${CC}(\theta ,\Delta x,\Delta y)={\sum }_{x}{\sum }_{y}A\left(x,y\right)B^{\prime} (x+\Delta x,y+\Delta y,\theta )$$
  where $A\left(x,y\right)$ is the intensity of the reference image, and $B^{\prime} (x\,+{{\Delta }}x,\,y\,+{{\Delta }}y,\theta )$ is the intensity of the spinal image after applying a rotation θ and a translation (Δx, Δy). The registration was performed on the B0 images, which provide consistent anatomical signal across the brain and spinal cord. The transformation matrices resulting from this step were subsequently applied to the corresponding diffusion volumes to ensure accurate alignment of the DTI data across the brain and spine.
5. After this, seven different rotation angles were tested, ranging from -12° to 12°, and the angle with the highest correlation was selected. The optimal translation was then estimated using the method described above.
6. To assess the quality of spatial alignment between the cranial and spinal diffusion-weighted imaging acquisitions, we used the derived fractional anisotropy (FA) maps as a common three-dimensional representation of white matter microstructure. For each pair of FA volumes, corresponding two-dimensional slices were extracted and binarized using a fixed FA threshold of 0.35, which isolates regions with high anisotropy typically associated with white matter tracts. To focus the comparison on anatomically meaningful structures and reduce the influence of noise, we retained only the largest connected component that was closest to the geometric center of the image. The spatial overlap between the resulting binary masks was quantified using the Dice similarity coefficient, defined as:
  $${{{\rm{Dice}}}}({{{\rm{A}}}},{{{\rm{B}}}})=\frac{2{{{\rm{| }}}}{{{\rm{A}}}}\cap {{{\rm{B}}}}{{{\rm{| }}}}}{{{{\rm{| }}}}{{{\rm{A}}}}{{{\rm{| }}}}+{{{\rm{| }}}}{{{\rm{B}}}}{{{\rm{| }}}}}$$
  where A and B denote the binary masks extracted from the fixed and moving images, respectively. A Dice score of 1 indicates perfect spatial correspondence, while a score of 0 indicates no overlap. This procedure allows us to evaluate the local registration quality in a robust and anatomically relevant manner.

**Table 1:** ROI design

Tracts	ROI placement [reference numbers]
Corticospinal	Pyramid of the medulla oblongata [28, 32]
Rubrospinal	Mediocaudal part of the red nucleus [29, 32]
Spinocerebellar	Ventral: superior cerebellar peduncles [30, 32] Dorsal: inferior cerebellar peduncles [30, 32]
Spinothalamic	VPL nucleus of the thalamus, with region of avoidance on dorsal columns [32]
Dorsal columns	VPL nucleus of the thalamus and gracilis and cuneatus columns at the lower part of the medulla [31, 32]

*ROI* Region of interest, *VPL* Ventro-postero-lateral

#### Region of interest (ROI) design (Table 1)

For motor tracts, the pyramid (of the medulla oblongata) [28] was defined as the ROI for the corticospinal tract, and the mediocaudal part of the red nucleus [29] was defined as the ROI for the rubrospinal tract. For sensitive tracts, the superior and inferior cerebellar peduncles were defined as the ROIs for the ventral and dorsal spinocerebellar tracts [30], respectively. The ventral posterolateral (VPL) nucleus [31] was defined as the ROI for both the spinothalamic tract and the dorsal columns, while the gracilis and cuneatus nuclei were defined as the ROI for the dorsal columns only. To specifically visualize the spinothalamic tract only, the VPL nucleus was defined as a ROI, and the gracilis and cuneatus nuclei were defined as a region of avoidance [32]. ROI were drawn on T2-weighted images, then automatically merged with diffusion-weighted images (using DSI Studio).

#### Fiber tracking

Tractography was performed using the DSI Studio software package [33]. DSI Studio provides a deterministic fiber-tracking algorithm based on a generalized q-sampling imaging approach [34], which improves accuracy [33]. The deterministic method was chosen because, in the spinal cord, white matter fibers are predominantly parallel with minimal crossing, making this approach the reference standard. Moreover, a previous study demonstrated that deterministic tractography yields longer, more continuous fiber tracts with fewer premature terminations compared to probabilistic methods, supporting its suitability for spinal cord tractography [5]. The tractography algorithm used the following parameters: step size = 0.1 mm, fiber length = 10 to 1,000 mm, angular threshold = 90°, and an adaptive fractional anisotropy threshold. This fractional anisotropy threshold was selected using a compromise value between maximum anatomical details and minimum “noise.” A total of 1,000,000 tracts were generated, using DSI Studio’s default “seed = auto” option, which automatically distributes seed points within the defined ROI based on a 0.5 seed-per-voxel ratio. Fibers were displayed as 0.10-mm diameter tubes. The mean fiber length was then extracted as a key metric to assess the stitching outcomes. This choice was made because fiber length reflects the continuity and coherence of fibers within each field of view, allowing to evaluate the accuracy and effectiveness of the stitching process in maintaining the anatomical integrity of neural tracts across multiple FOV. Longer and uninterrupted fiber tracts will indicate successful alignment of adjacent FOV.

Then, automatic registration was performed between DTI and T2-weighted imaging (allowing tractography to be superimposed on T2-weighted imaging), using the “registration” tool in DSI Studio. Once each tract of interest (*i.e*., corticospinal tract, rubrospinal tract, spinocerebellar tracts, spinothalamic tract, and dorsal columns) was highlighted and merged on T2-weighted imaging, an individual atlas of the spinal cord white matter was drawn, taking the C5 level of the spine as reference.

## Results

### Stitching

**Fig. 2:**
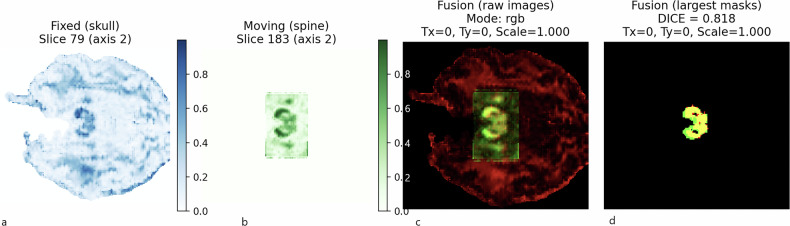
Workflow for evaluating the quality of brain–spinal cord DTI image fusion using the Dice similarity coefficient. (**a**) FA map of the brain, (**b**) FA map of the spinal cord, (**c**) fused FA dataset following multimodal registration, (**d**) binary FA map obtained after thresholding at 0.35, used to compute the spatial overlap between regions of interest. The Dice similarity coefficient was then calculated to quantify the alignment accuracy between the brain and spinal cord FA structures

The Dice similarity coefficients obtained to assess the quality of the fusion between brain and spinal cord DTI datasets ranged from 0.795 to 0.818, suggesting that the proposed fusion approach yields consistent and reliable alignment of diffusion-based structures along the neuroaxis (Fig. 2). The median length of fibers from the stitched images (192 mm) was significantly higher compared to the median length of fibers from the brain FOV (111.5 mm) and to the median length of fibers from the spinal cord FOV (115 mm, Table 2).

**Table 2:** Dice similarity coefficient and mean fiber length

Subject number, sex, age (years)	Dice similarity coefficient	Median fiber length (mm)		
		Within the “brain FOV”	Within the “spinal cord FOV”	Within the stitched images
Subject 1, female, 37	0.795	102	126	180
Subject 2, female, 23	0.809	108	110	195
Subject 3, male, 35	0.818	127	100	189
Subject 4, male, 35	0.812	115	120	197
Median	0.8105	111.5	115	192

*FOV* Field of view

### Descending motor tracts

**Fig. 3:**
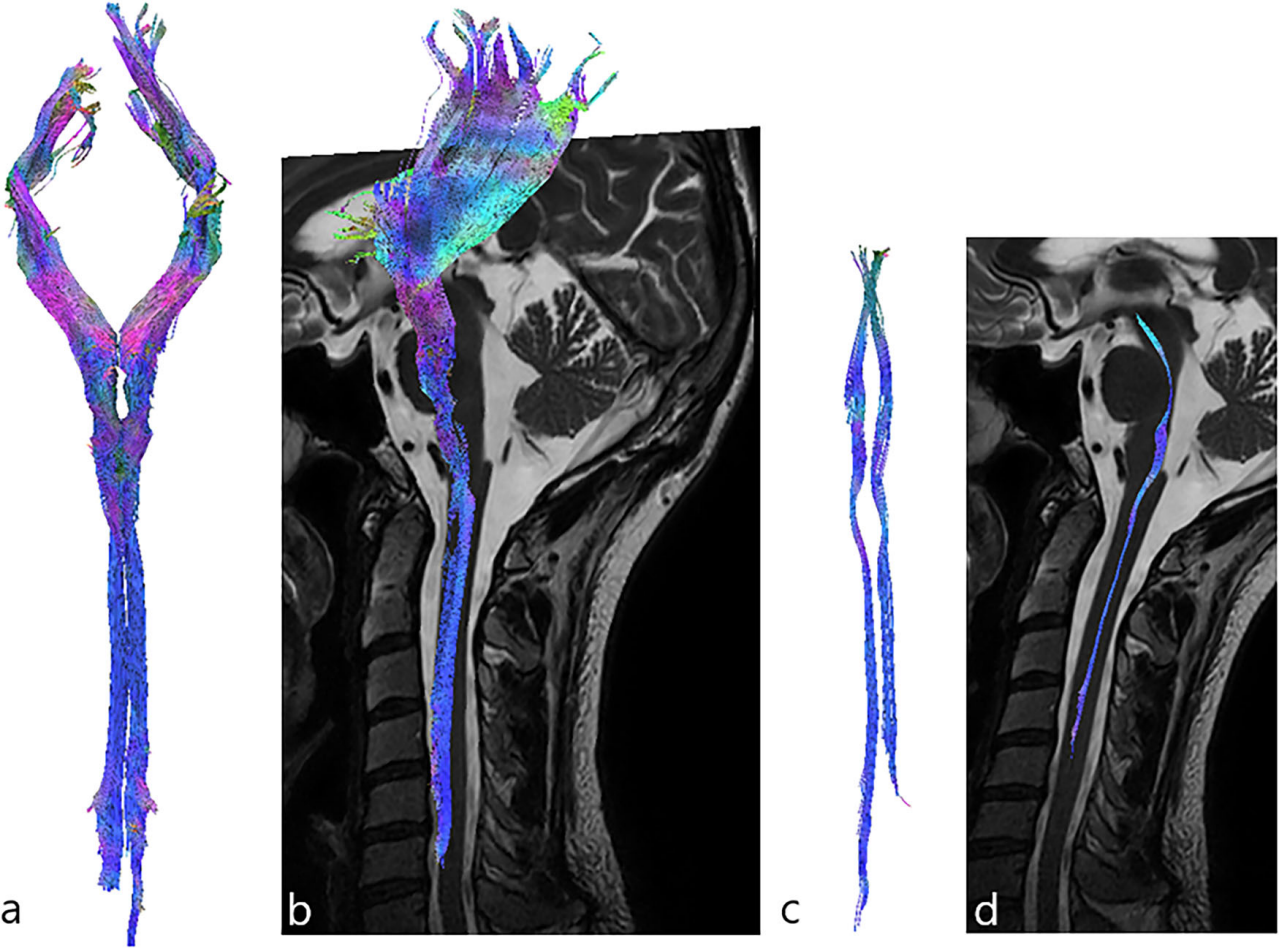
3D-tractography rendering of motor pathways. **a** Coronal view of the corticospinal tract. **b** Sagittal view of the corticospinal tract, merged on sagittal T2-weighted images. **c** Coronal view of the rubrospinal tract. **d** Sagittal view of the rubrospinal tract merged on sagittal T2-weighted images

The corticospinal tract was the largest descending spinal tract. It originated from the cerebral cortex, passing through the corona radiata, internal capsule and pyramids (where ROIs were drawn). The corticospinal tract then crossed the midline and descended into the lateral funiculus of the spinal cord. Notably, tracked corticospinal fibers were also represented in the intermediate gray matter and the entire ventral horn (*i.e*., lamina VII, VIII, and IX according to the gray matter segmentation performed by Rexed [35], Fig. 3a, b).

The rubrospinal tracts originated from the mediocaudal magnocellular part of the red nucleus (drawn as a ROI). These fibers crossed the median raphe in the ventral tegmentum decussation and descended in the dorsal part of the brainstem. Finally, the rubrospinal tracts were found in the lateral funiculus of the spinal cord. Some fibers were also found in the lateral part of the ventral horn. It is important to note that some fibers terminated at the middle cervical levels, although the FOV included the lower cervical levels (Fig. 3c, d).

### Ascending sensitive tracts

**Fig. 4:**
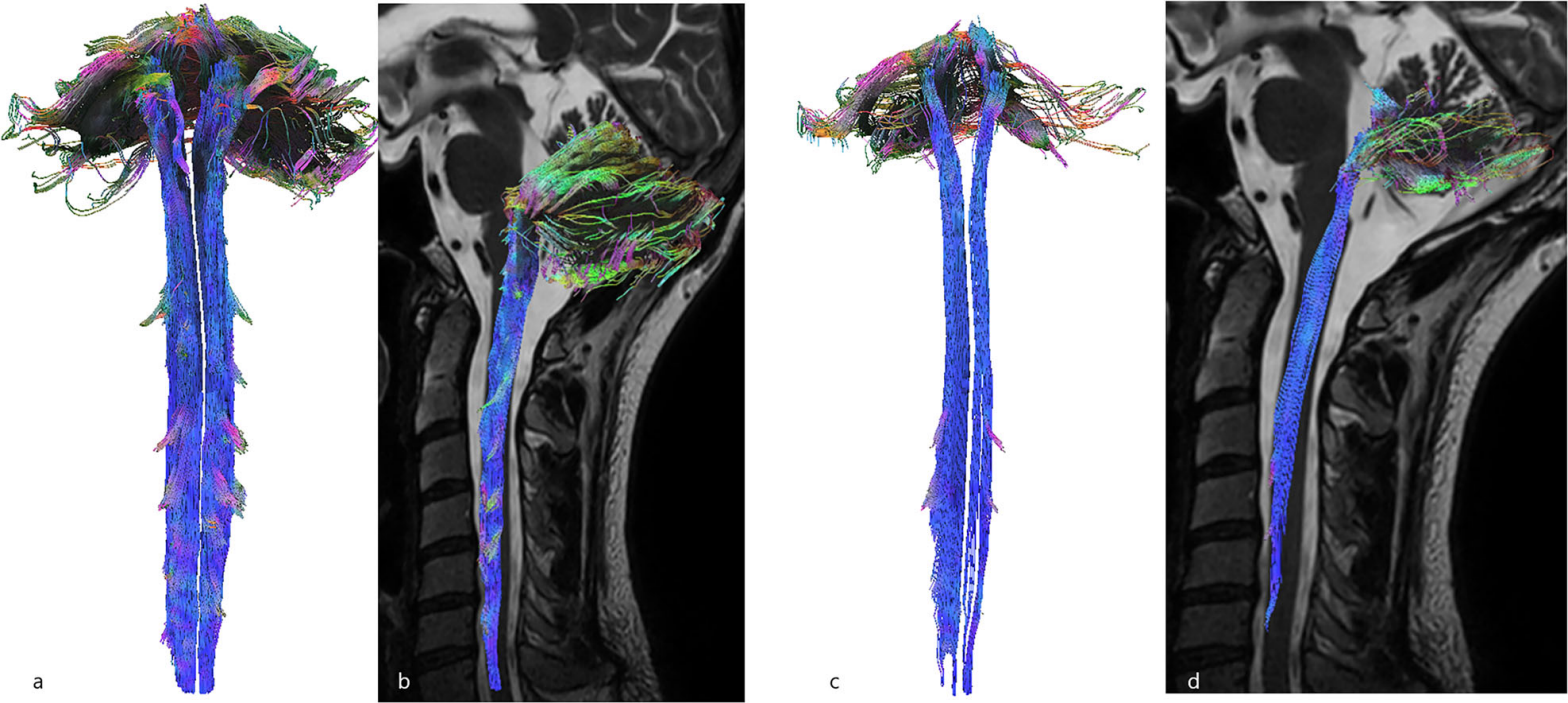
3D-tractography rendering of the spinocerebellar tracts. **a** Coronal view of the dorsal spinocerebellar tract. **b** Sagittal view of the dorsal spinocerebellar tract merged on sagittal T2-weighted images. **c** Coronal view of the ventral spinocerebellar tract. **d** Sagittal view of the ventral spinocerebellar tract merged on sagittal T2-weighted images

The dorsal spinocerebellar tracts formed a double-arch in the lateral part of the spinal cord, but were more superficial and larger than the ventral spinocerebellar tracts. Fibers passed through the inferior cerebellar peduncles and terminated in both cranial and caudal parts of the vermis. The dorsal spinocerebellar tracts were located superficially, in the dorsolateral part of the dorsal funiculus and the lateral funiculus of the spinal cord (Fig. 4a, b).

The ventral spinocerebellar tracts also formed a double-arch in the lateral part of the spinal cord and ascended to the superior cerebellar peduncles. A few crossing fibers were observed at the cerebellum level. The ventral spinocerebellar tracts were found in the dorsolateral part of the dorsal funiculus and the lateral funiculus of the spinal cord (Fig. 4c, d).

**Fig. 5:**
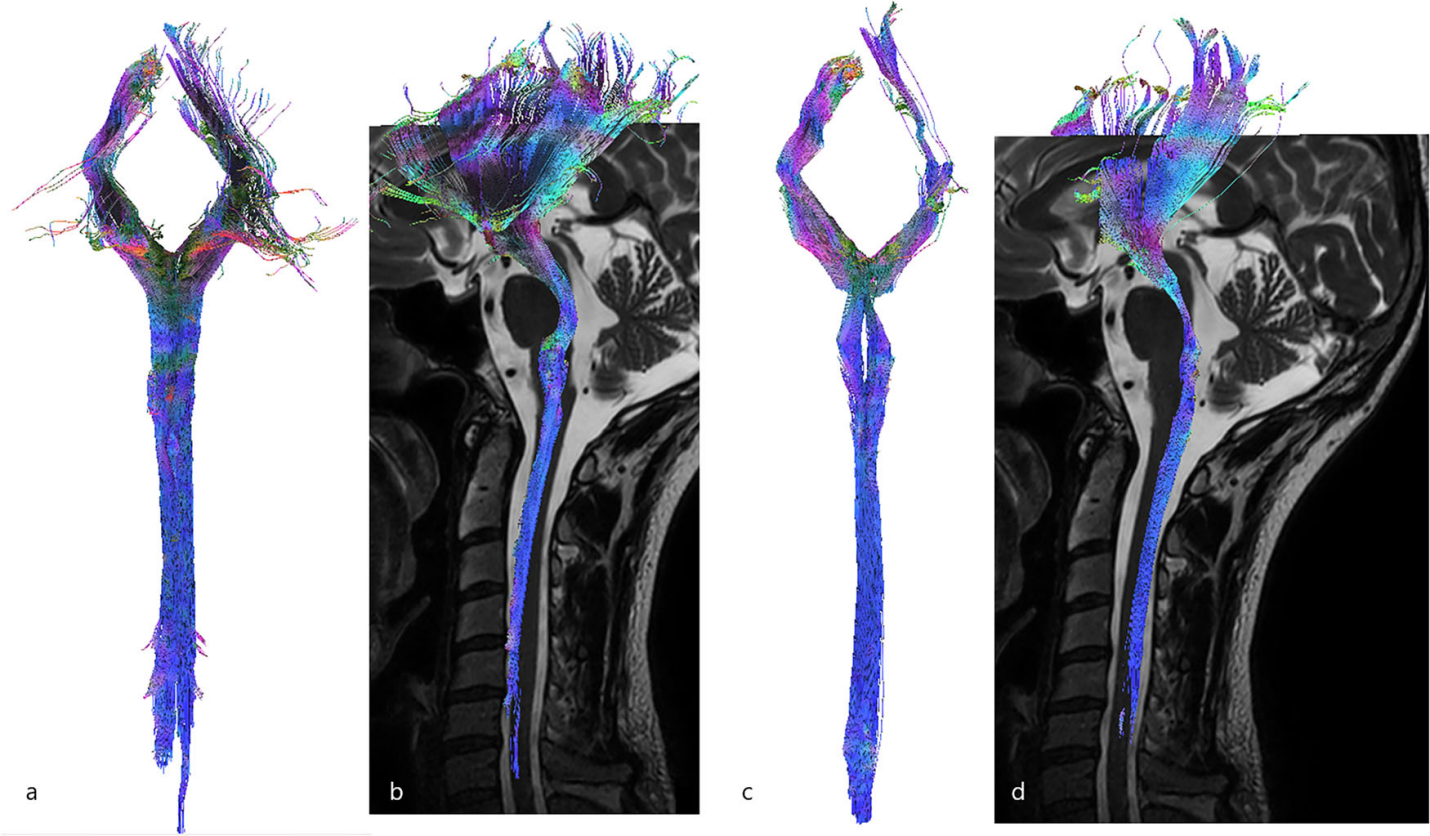
3D-tractography rendering of the sensitive pathways. **a** Coronal view of the spinothalamic tract. **b** Sagittal view of the spinothalamic tract merged on sagittal T2-weighted images. **c** Coronal view of the dorsal columns. **d** Sagittal view of the dorsal columns tracts merged on sagittal T2-weighted images

The spinothalamic tracts ascended ventrolaterally, forming an indentation at the dorsal part of the pons before joining the VPL nucleus of the thalamus. The spinothalamic tracts were found in the dorsal horn (*i.e*., lamina I, II, III, IV, and V according to the gray matter segmentation of Rexed [35]), in the ventromedial part of the ventral funiculus and the ventral part of the lateral funiculus of the spinal cord (Fig. 5a, b).

The dorsal columns ascended in the dorsal part of the spinal cord to the gracilis and cuneatus fasciculi (which were drawn as ROI in the dorsal part of the medulla) and were positioned more medially and ventrally to form the medial lemniscus. Finally, the somatosensory tracts took a ventral direction toward the ventro-postero-lateral nucleus of the thalamus. Gracilis and cuneatus tracts were located in the dorsal funiculus of the spinal cord and left a lateral notch for the spinocerebellar fibers (Fig. 5c, d).

### Spinal cord atlas

**Fig. 6:**
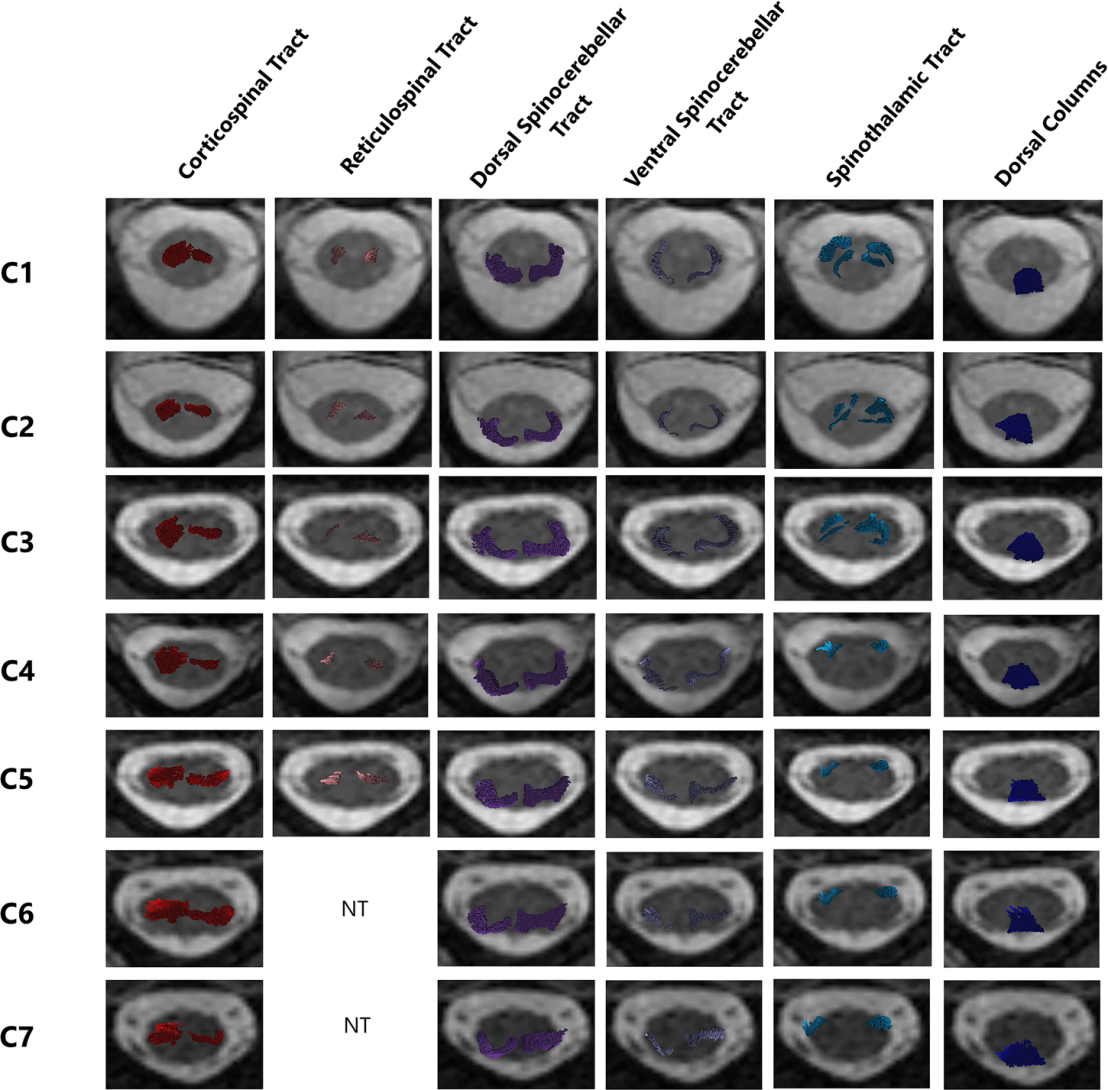
Spinal cord atlas. Each tract is merged on axial T2-weighted images at each cervical spine level. NT, Not tracked

**Fig. 7:**
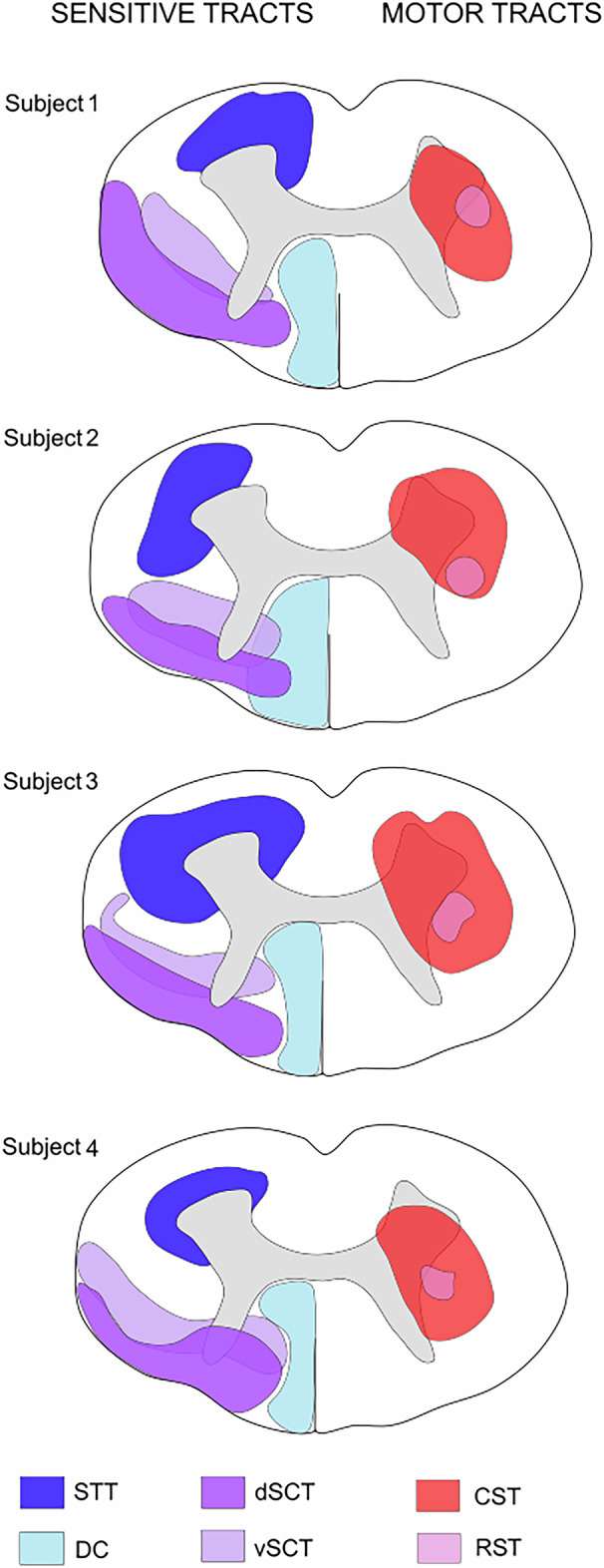
Inter-individual variability of spinal cord anatomy. Representation of spinal cord atlases in each healthy subject, at the C5 spine level. CST, Corticospinal tract; DC, Dorsal columns; dSCT, Dorsal spinocerebellar tract; RST, Rubrospinal tract; STT, Spinothalamic tract; vSCT, Ventral spinocerebellar tract

Although the location of spinal tracts varied among the four healthy subjects analyzed herein, demonstrating the interindividual variability of the spinal tract anatomy, there was notable reproducibility in the anatomy of spinal tracts between subjects and spine levels (Fig. 6). The dorsal columns were the most reproducible. Although the dorsal spinocerebellar tract was more superficial and dorsal than the ventral spinocerebellar tract, these tracts overlapped in the lateral funiculus of the spinal cord. The spinothalamic tract was more variable, even when located in the ventrolateral part of the spinal cord, around the ventral horn. Additionally, some spinothalamic fibers were tracked into the dorsal horn. The location of the corticospinal tract was challenging to reproduce between subjects, but it primarily occupied the ventromedial part of the lateral funiculus and the ventral horn. The rubrospinal tract was consistently located in the lateral funiculus between the dorsal and ventral horns, and overlapped with the corticospinal tract (Fig. 7).

## Discussion

The present proof-of-concept study found that stitching between brain and spinal cord DTI volumes is possible, and supports the robustness of the proposed pipeline. This approach allows the tracking of projection tracts (*i.e*., spinal tracts) and the production of an *in vivo* atlas of the spinal cord white matter.

### Stitching on corrected images *versus* tensors or neural fibers

In the present study, a cross-correlation registration methodology based on B0 was employed to stitch corrected images of the brain and spinal cord DTI volumes, favoring the enhancement of overall anatomical alignment. This approach offers several advantages over stitching directly on tensors or tractography fibers. First, B0 images provide consistent anatomical contrast and are not affected by directional noise, facilitating robust alignment through cross-correlation. Additionally, this method reduces the propagation of errors that may arise from the initial tensor estimation or tractography [36], providing a more stable basis for further analysis. Unlike tensor-based stitching, which would require complex reorientation and interpolation steps to preserve directional integrity, our approach offers a simpler yet effective solution. Nevertheless, this approach has some limitations. It may have overlooked subtle but crucial variations in tensor orientations and fiber trajectories that are better captured when operating directly on tensors or fibers (from the tractography) [37]. Stitching on original diffusion tensors might preserve tensor-specific information that could be lost during image fusion, potentially improving the continuity of fiber tracking across stitched regions. While this preserved directional information, it requires complex tensor reorientation and interpolation processes. Another approach consists of stitching on tractography fibers, which can provide detailed integration of neural pathways but is highly sensitive to the accuracy of the tractography algorithm and initial diffusion data quality, and potentially may suffer from the propagation of inherent errors in the fiber-tracking process.

### Cross-correlation *versus* scale-invariant feature transform (SIFT)

The cross-correlation registration methodology enhanced alignment accuracy between DTI datasets by maximizing the similarity of the corresponding regions [19], thus providing a more coherent representation of neural pathways. Cross-correlation is effective when intensity patterns are consistent, offering robust performance for aligning images with similar contrast and texture characteristics [19, 38]. However, it may struggle with larger anatomical distortions or regions with low signal-to-noise ratios, potentially impacting the precision of the stitching process. Moreover, this method was computationally intensive (about 12 h), as reported elsewhere [39], requiring substantial processing time and resources. In contrast, SIFT identifies and matches key features across images, regardless of scale, rotation, or illumination changes, making it versatile for a wide range of imaging conditions [40, 41]. However, when we used SIFT for the stitching of the DTI images used herein, this failed to detect and match similar local features, due to the low spatial resolution in DTI images, which was further compounded by low signal-to-noise ratio even after distortion corrections (data not shown).

### Spinal cord atlas

The main strength of the present study is the production of an *in vivo* healthy human spinal cord atlas. By stitching brain and spinal cord image volumes, a white matter atlas of the spinal cord was built without prior assumptions, drawing ROIs for each tract of interest at the brainstem level (*i.e*., outside of the spinal cord). This process ensures quality control of each projection tract by following its fibers at the brain level and verifying its anatomical cerebral location, compared to existing brain atlases.

The present study offers accurate mapping of spinal tracts, *in vivo*, in humans. Conversely, existing atlases of spinal projection tracts were constructed using animal models, involving rodents and non-human primates, by employing invasive validation techniques, such as tracing pathways and histological verification [42, 43], which are not feasible in humans. In addition, extrapolation of animal data to the human spinal cord poses challenges due to species-specific differences in anatomy and physiology.

Beyond its methodological and anatomical contributions, the study herein carries important clinical implications. First, by providing a detailed and individualized mapping of spinal tracts, it enhances our understanding of interindividual variability, particularly concerning the distribution of white matter pathways. This interindividual variability is critical for patient-specific approaches in patients with neurological disorders affecting the spinal cord.

The ability to generate subject-specific tractography through an individualized stitching process opens new perspectives for personalized medicine, allowing clinicians to tailor therapeutic strategies based on each patient’s unique spinal cord anatomy. Furthermore, this refined spinal cord atlas could serve as a valuable tool for neurosurgical planning, especially in procedures requiring precise localization of spinal tracts, such as intramedullary tumors [15]. It may also contribute to improving the accuracy of prognostic assessments in SCI patients, or other neurological pathologies as multiple sclerosis [44] or degenerative myelopathy [45], by providing insights into tract integrity and connectivity, thus guiding treatment strategies. In this way, future studies including patients with central nervous system disorders will be essential to assess the clinical applicability and diagnostic relevance of this method.

### Limitations and future directions

Despite the advancements achieved through cross-correlation registration methodology, this study faced some limitations. Although this study is a proof of concept, the sample size was relatively small, potentially limiting the generalizability of the spinal cord atlas reported herein to the general population, and these results should be considered preliminary. Validation on a larger cohort will be essential to confirm the reproducibility, accuracy, and generalizability of the proposed method. The cross-correlation methodology was highly dependent on the quality of diffusion images [39, 46]. Despite using advanced distortion correction techniques, such as FSL topup and eddy [7, 27], residual artifacts can still impact image alignment accuracy. This dependency may limit the precision of the stitching process, especially when the spinal cord and brainstem images have varying signal-to-noise ratios. Additionally, the computational intensity of the cross-correlation process necessitated significant processing resources and time [38, 39], which can be limiting factors in large-scale studies. This highlights the need for more efficient algorithms and computational strategies to expedite registration, especially using neural networks and deep learning.

One promising perspective for future research lies in achieving a seamless continuity of tractography across the entire central nervous system, encompassing both the brain and the full spinal cord. However, achieving this goal will require addressing several challenges, particularly the need for multiple FOV acquisitions at the spinal cord level. The thoracic spinal cord poses significant constraints for DTI acquisition due to its anatomical and physiological characteristics [6, 47], and successfully performing DTI stitching between spinal cord segments remains an underexplored area [48]. Overcoming these barriers will likely be the first key to advancing this methodology to achieve whole central nervous system tractography.

Finally, while this study proposed a personalized mapping of the spinal cord, this could also be perceived as a limitation. To generalize the present results to the broader population, fusing diffusion images from healthy subjects would represent a significant step toward a “spinal cord connectome.” Our team is currently working on this by spatially normalizing individual atlases into a common template space to generate a population-based atlas.

In conclusion, this study introduces a novel stitching process between brain and spinal cord DTI, enabling accurate tracking of projection tracts and the creation of an *in vivo* white matter atlas of the spinal cord. It contributes to the advancement of spinal cord anatomy and paves the way for personalized medicine in the field of spinal cord diseases.

## Data Availability

On request, and under certain conditions, the data presented herein can be shared for scientific purposes.

## Abbreviations

DSI: Diffusion spectrum imaging
DTI: Diffusion tensor imaging
FA: Fractional anisotropy
FOV: Field of view
FSL: Functional magnetic resonance imaging of the brain Software Library
MRI: Magnetic resonance imaging
ROI: Region of interest
SIFT: Scale-invariant feature transform
VPL: Ventral posterolateral
